# Mycorrhizal Associations and Trophic Modes in Coexisting Orchids: An Ecological Continuum between Auto- and Mixotrophy

**DOI:** 10.3389/fpls.2017.01497

**Published:** 2017-08-29

**Authors:** Hans Jacquemyn, Michael Waud, Rein Brys, Félix Lallemand, Pierre-Emmanuel Courty, Alicja Robionek, Marc-André Selosse

**Affiliations:** ^1^Plant Conservation and Population Biology, Department of Biology, KU Leuven Leuven, Belgium; ^2^Research Institute for Forest and Nature Geraardsbergen, Belgium; ^3^Institut de Systématique, Évolution, Biodiversité, UMR 7205, CNRS, MNHN, UPMC, EPHE, Muséum National d’Histoire Naturelle, Sorbonne Universités Paris, France; ^4^Department of Biology, University of Fribourg Fribourg, Switzerland; ^5^The Laboratory of Freshwater Ecology, Department of Plant Ecology, University of Gdańsk Gdańsk, Poland; ^6^Department of Plant Taxonomy and Nature Conservation, University of Gdańsk Gdańsk, Poland

**Keywords:** carbon nutrition, coexistence, mycoheterotrophy, orchids, partial mycoheterotrophy, rhizoctonia

## Abstract

Two distinct nutritional syndromes have been described in temperate green orchids. Most orchids form mycorrhizas with rhizoctonia fungi and are considered autotrophic. Some orchids, however, associate with fungi that simultaneously form ectomycorrhizas with surrounding trees and derive their carbon from these fungi. This evolutionarily derived condition has been called mixotrophy or partial mycoheterotrophy and is characterized by ^13^C enrichment and high N content. Although it has been suggested that the two major nutritional syndromes are clearly distinct and tightly linked to the composition of mycorrhizal communities, recent studies have challenged this assumption. Here, we investigated whether mycorrhizal communities and nutritional syndromes differed between seven green orchid species that co-occur under similar ecological conditions (coastal dune slacks). Our results showed that mycorrhizal communities differed significantly between orchid species. Rhizoctonia fungi dominated in *Dactylorhiza* sp., *Herminium monorchis*, and *Epipactis palustris*, which were autotrophic based on ^13^C and N content. Conversely, *Liparis loeselii* and *Epipactis neerlandica* associated primarily with ectomycorrhizal fungi but surprisingly, ^13^C and N content supported mixotrophy only in *E. neerlandica*. This, together with the finding of some ectomycorrhizal fungi in rhizoctonia-associated orchids, suggests that there exists an ecological continuum between the two syndromes. The presence of a large number of indicator species associating with individual orchid species further confirms previous findings that mycorrhizal fungi may be important factors driving niche-partitioning in terrestrial orchids and therefore contribute to orchid coexistence.

## Introduction

Since the early discoveries that orchids significantly rely on mycorrhizal fungi for seed germination and seedling establishment (Bernard, 1899; Burgeff, 1909), it has become increasingly clear that mycorrhizal fungi play an important role in carbon nutrition of orchids and therefore also in their population dynamics and spatial distribution (McCormick and Jacquemyn, 2014). In the early stages of orchid development, fungi provide carbon to orchid seeds, which due to their tiny size lack the necessary nutritional resources to develop (Smith and Read, 2008; Dearnaley et al., 2016). Although several orchid species belonging to various, unrelated genera remain achlorophyllous at adulthood (Merckx, 2013) and maintain a continual reliance on fungal carbon (a nutrition called mycoheterotrophy), most orchids are green at adulthood and become photosynthetic after the first green leaves have developed. However, the extent to which mycorrhizal fungi contribute to carbon nutrition of adult green orchids remains unclear.

In general, two main nutritional syndromes have been described for adult green orchids (Selosse et al., 2016). In some green orchid species, often related to the above-mentioned achlorophyllous species, a partly heterotrophic syndrome has been described, called partial mycoheterotrophy or mixotrophy (Selosse and Roy, 2009; Hynson et al., 2013). Such species often grow in closed forest habitats with limited light-availability and associate with the same fungal guilds as achlorophyllous species, most often fungi that form ectomycorrhizae on surrounding trees (Bidartondo et al., 2004; Selosse et al., 2004). Direct evidence for mixotrophy comes from three observations. First, such orchids display reduced photosynthetic ability either due to intrinsic limitation (Girlanda et al., 2006; Cameron et al., 2008) or to environmental conditions (Julou et al., 2005). Secondly, achlorophyllous variants can survive in nature (Roy et al., 2013; Shefferson et al., 2016). Third, these orchids have ^13^C and ^15^N natural abundances and total N content that are intermediate between those of autotrophic plants and achlorophyllous orchids (Abadie et al., 2006; Hynson et al., 2013, 2016). The latter are highly enriched in both isotopes and have a high N content (Trudell et al., 2003; Hynson et al., 2016), likely because similar features characterize the biomass of the ectomycorrhizal fungi supporting them (Hynson et al., 2013). Thus, mixotrophic orchids combine photosynthesis and heterotrophic nutrition on ectomycorrhizal fungi.

The second syndrome, called autotrophy, concerns the vast majority of orchids that associate with basidiomycetous fungi collectively called ‘rhizoctonias.’ Rhizoctonia fungi belong to Tulasnellaceae, Ceratobasidiaceae (two families of the order Cantharellales), and Serendipitaceae (Sebacinales; Dearnaley et al., 2012) and behave as saprotrophs in the soil or as endophytes in non-orchid plants (Girlanda et al., 2011; Selosse and Martos, 2014). Based on *in vitro* evidence of mycorrhizal C flow observed in one species (Cameron et al., 2008), and on the fact that rhizoctonia-associated orchids do not show unusual ^13^C enrichment, these orchids are usually considered autotrophic. Yet, this view has recently been questioned based on data that suggested orchid incorporation of organic matter issuing from a rhizoctonia source. Firstly, ‘autotrophic’ orchids still have minor deviations, either positive or negative, in ^13^C abundance as compared to autotrophic species, coupled with high ^15^N enrichment and high N content (Hynson et al., 2013; Selosse and Martos, 2014). Secondly, they display higher ^2^H enrichment than autotrophic plants, a feature that may be explained if orchids receive ^2^H-rich organic matter from their mycorrhizal fungi (Gebauer et al., 2016). Yet, despite evidence that rhizoctonia fungi are widespread orchid partners that support the nutrition during germination, they never fully support achlorophyllous adult orchids at maturity (see review in Dearnaley et al., 2012). Whenever a species from one of the families of rhizoctonia fungi was found to support full mycoheterotrophy, it belonged to species that by exception had ectomycorrhizal abilities (e.g., Bougoure et al., 2010; Yagame et al., 2012). Thus, the amount of C provided by rhizoctonia fungi to orchids may be limited by their specific saprotrophic or endophytic ecology (Selosse and Martos, 2014) and the occurrence of mixotrophy in rhizoctonia-associated orchids remains debated.

It has been suggested that the emergence of substantial levels of heterotrophy detectable by ^13^C abundance is linked to a shift from rhizoctonia fungi, the ancestral orchids’ partners, to ectomycorrhizal fungi, and that this shift has occurred frequently in orchid evolution (Hynson et al., 2013). Some orchid genera contain species with both types of nutritional syndrome and therefore support evidence for this evolutionary shift. Typical examples are species within *Cymbidium* (Ogura-Tsujita et al., 2012) or *Neottia* (Těšitelová et al., 2015). However, with the use of next-generation sequencing that considerably enhanced the depth of detection of fungal communities (Waud et al., 2014), there is increasing evidence that some ectomycorrhizal fungi can occur in rhizoctonia-associated orchids, and vice-versa (Jacquemyn et al., 2014, 2016a; Oja et al., 2015; Těšitelová et al., 2015). Clearly, this challenges the distinction between the two syndromes in terms of associated fungi.

In this study, we investigated the relationship between nutritional syndromes and the composition of mycorrhizal communities in a range of terrestrial green orchids that commonly co-occur in wet dune slacks. Dune slacks are linear depressions that lay close to sea level in coastal dune systems and are characterized by high irradiance levels. They often undergo a successional series. Young dune slacks are generally species-poor (Bossuyt et al., 2003), but as they age, the vegetation cover increases toward completion and a true soil begins to develop. Previous molecular investigations have shown that dune slacks contain very diverse mycorrhizal communities in the soil (Geml et al., 2014). More specifically, to assess whether each orchid species from dune slacks could be assigned to a definite fungal community and nutritional syndrome, we combined detailed assessments of the mycorrhizal communities associating with each orchid species with isotopic studies. Finally, we investigated the correlation between the dominant type of mycorrhizal fungi and the level of heterotrophy as detected by ^13^C and N abundance.

## Materials and Methods

### Study System

Molecular and isotopic data were collected from seven orchid species that co-occurred in two coastal dune slacks along the Belgian coast (Ter Yde: 51°8′18″ N – 2°42′14″ E; Westhoek: 51°5′16″ N – 2°33′48″ E). Both sampling sites were devoid of trees and had similar, high light conditions. The sampled orchid species included two *Epipactis* species (*E. neerlandica* and *E. palustris*), three *Dactylorhiza* species (*D. fuchsii, D. incarnata*, and *D. praetermissa*), *Herminium monorchis*, and *Liparis loeselii*.

### Mycorrhizal Sampling and Analyses

To assess differences in orchid mycorrhizal communities between the seven species, young roots of five replicate individuals were collected for each species in the most central part of each of the study populations in summer 2015. After roots were carefully excavated from the soil, they were transported to the laboratory and immediately surface sterilized (30 s submergence in 1% sodium hypochlorite, followed by three 30 s rinse steps in sterile distilled water). Mycorrhizal association was confirmed by detection of peloton structures using thin cross sections of orchid roots. These sections were gently cleared in hot alkali, stained using Trypan blue, and viewed with interference contrast microscopy (Brundrett et al., 1990). Subsequently, DNA was extracted from 0.5 g mycorrhizal root fragments using the UltraClean Plant DNA Isolation Kit as described by the manufacturer (Mo Bio Laboratories, Inc., Solana Beach, CA, United States) and 10 times diluted afterward. Amplicon libraries were created using the broad-spectrum internal transcribed spacer (ITS) primers ITS3 (5′-GCATCGATGAAGAACGCAG-3′) and ITS4OF (5′-TTACTAGGGGAATCCTTGTT-3′) (White et al., 1990; Taylor and McCormick, 2008). This primer pair has been shown to amplify substantial diversity of sequences and to detect a variety of orchid-associating mycorrhizal families (Waud et al., 2014). All samples (65 in total) were assigned unique MID (Multiplex Identifier) barcode sequences according to the guidelines for 454 GS-FLX Lib-L sequencing.

Polymerase chain reaction (PCR) amplification was performed in duplicate in a 25 μl reaction volume containing 0.15 mM of each dNTP, 0.5 μM of each primer, 1 U Titanium Taq DNA polymerase, 1X Titanium Taq PCR buffer (Clontech Laboratories, Palo Alto, CA, United States), and 1 μl of a 10-times diluted DNA extract. PCR conditions were as follows: initial denaturation of 2 min at 94°C followed by 30 cycles of 45 s at 94°C, 45 s at 59°C, and 45 s at 72°C. After resolving the amplicons by agarose gel electrophoresis, amplicons within the appropriate size range (∼250–500 bp) were cut from the gel and purified using the Qiaquick gel extraction kit (Qiagen, Hamburg, Germany). Purified dsDNA amplicons were quantified using the Qubit fluorometer (Invitrogen) and pooled in equimolar quantities of 1.00E+10 molecules per sample. This yielded two amplicon libraries, each representing one of the two PCR replicates. The quality of the amplicon libraries was assessed using an Agilent Bioanalyzer 2100 and high sensitivity DNA chip (Agilent Technologies, Waldbronn, Germany). Each amplicon library was loaded onto 1/8th of a 454 Pico Titer Plate (PTP). Pyrosequencing was performed using the Roche GS FLX instrument and Titanium chemistry according to the manufacturer’s instructions (Roche Applied Science, Mannheim, Germany).

### Stable Isotope Analyses

Samples for isotopic analysis were collected from the same set of orchids at Ter Yde and Westhoek in summer 2015, at the same time that roots were collected for mycorrhizal analyses. At Ter Yde, we sampled *Epipactis palustris, E. neerlandica* and *Dactylorhiza praetermissa*, and at Westhoek, in addition to the two first previous species, we sampled *Dactylorhiza fuchsii, D. incarnata, H. monorchis*, and *L. loeselii*. At both sites, we also sampled representatives of several surrounding non-orchid autotrophic plants (see names in **Figure 4**). For each species, we collected five leaves, each from different plants. Samples were ground in 2 mL Eppendorf tubes in a ball mill (MM200, Retsch Gmbh, Haan, Germany) and analyzed for total N concentration, as well as ^13^C/^12^C and ^15^N/^14^N ratios using an elemental analyser (EA) coupled to a ThermoFinnigan DeltaV Advantage Continuous-Flow Isotope-ratio mass spectrometer, and expressed as δ-values (Hynson et al., 2013). Isotope values were calibrated using internal calibrated standards (EDTA and ammonium oxalate). The standard deviations of the replicated standard samples were 0.029‰ for ^13^C and 0.215‰ for ^15^N.

### Data Analyses

#### Fungal Diversity

Sequences obtained from 454 pyrosequencing were assigned to the appropriate sample based on both barcode and primer sequences, allowing zero discrepancies, and were subsequently trimmed from the barcodes and primers using CUTADAPT 1.0 (Martin, 2011). Sequences were trimmed based on a minimum Phred score of 30 (base call accuracy of 99.9%) averaged over a 50 bp moving window. Sequences with ambiguous base calls or homopolymers longer than eight nucleotides were rejected, as were chimeric sequences detected by the UCHIME chimera detection program (*de novo* algorithm) (Edgar et al., 2011). Sequences which passed all quality control procedures were used as the basis for all further analyses. Minimum and maximum sequence lengths were set to 250 and 500 nucleotides, respectively. For further analysis, sequence data obtained for both PCR replicates were combined for each sample.

Operational taxonomic units (OTUs) were determined using UPARSE (Edgar, 2013), with sequences exceeding 97% sequence homology being clustered into the same OTU (Jacquemyn et al., 2015, 2016a,b; Oja et al., 2015). Global singletons and global doubletons (i.e., OTUs representing only one or two sequences in the whole dataset) were removed from further analysis since it has been shown that this improves the accuracy of diversity estimates (Ihrmark et al., 2012; Waud et al., 2014). The remaining OTUs were assigned taxonomic identities to the highest taxonomic rank possible based on BLAST (Altschul et al., 1990) results of representative sequences (as indicated by UPARSE) using GenBank (Benson et al., 2008), including uncultured/environmental entries. Finally, we manually screened OTUs for possible orchid-associating mycorrhizal families based on the data provided in Table 12.1 in Dearnaley et al. (2012) and information of previously detected mycorrhizal fungi from the roots, germinating seeds and protocorms of various *Epipactis* and *Dactylorhiza* species and *L. loeselii* (Kristiansen et al., 2001; Bidartondo et al., 2004; Selosse et al., 2004; Bidartondo and Read, 2008; Ogura-Tsujita and Yukawa, 2008; Ouanphanivanh et al., 2008; Shefferson et al., 2008; Illyés et al., 2009; Bailarote et al., 2012; Těšitelová et al., 2012; Jacquemyn et al., 2012, 2016b; Waud et al., 2017). Only OTUs corresponding to known orchid-associating mycorrhizal families were retained for further analysis. No previous data were available for *H. monorchis*.

#### Fungal Community Composition

Based on presence–absence data of the observed orchid mycorrhizal fungi in each of the sampled individuals, the fungal community composition associating with the different orchid species was visualized by non-metric multidimensional scaling (NMDS) using the Bray-Curtis coefficient as distance measure in the R software package ‘vegan’ (Oksanen et al., 2013). To test the hypothesis that the mycorrhizal communities differed between the sampled orchid species, we performed permutational analysis of variance (PERMANOVA; Anderson, 2001) using the ‘adonis’ function in the software package ‘vegan’ (Oksanen et al., 2013). Finally, Species Indicator Analysis was used to investigate whether mycorrhizal fungi could be identified that were significantly associated with one of the investigated orchid species. The ‘multipatt’ function in the R package ‘indicspecies’ (De Cáceres et al., 2010) was used to define indicator species of both individual species and combinations of species.

#### Isotope Signatures

For species found at two different sites, a Student’s *t*-test was used to test whether the mean δ^13^C or δ^15^N differed between sites. Analysis of variance (ANOVA) was used to evaluate differences in mean δ^13^C, δ^15^N, and % N among species from a given site. If the null hypothesis (no difference between means) was rejected, Tukey’s honestly significant difference (HSD) test was used to make pairwise multiple comparisons of the means. The alpha type I error threshold was set at 0.05. All statistical analyses were performed using the R environment for statistical computing (R Development Core Team, 2013).

## Results

### Mycorrhizal Diversity

The quality-filtered pyrosequencing data set comprised 556 fungal OTUs (89900 sequences), of which 104 (64732 sequences – 72.0%) were assigned to putatively orchid mycorrhizal OTUs according to Dearnaley et al. (2012) and information from previous studies that detected mycorrhizal fungi from the roots and protocorms of these and related orchid species (Supplementary Table S1). Individual samples contained on average 996 sequences of putatively orchid mycorrhizal OTUs, although there was substantial variation between samples (minimum: 101, maximum: 5489). Representative sequences for each mycorrhizal OTU found in this study were submitted to GenBank under the Accession Numbers KY083558 – KY083678 and MF567576 – MF567600.

**FIGURE 1:**
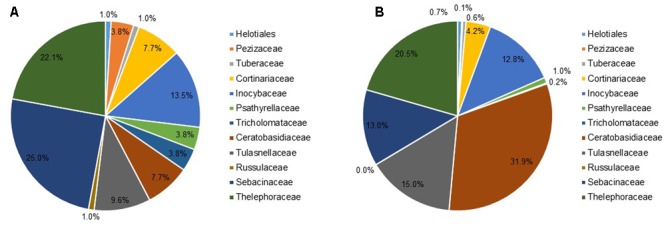
Frequency distribution of **(A)** the number of OTUs and **(B)** the number of sequences for the different mycorrhizal families found associating with the seven orchid species (*Dactylorhiza fuchsii, D. incarnata, D. praetermissa, Epipactis neerlandica, E. palustris, Herminium monorchis*, and *Liparis loeselii*) investigated in this study.

The majority of the detected fungi belonged to the Ceratobasidiaceae (8 OTUs, 20671 sequences) and Tulasnellaceae (10 OTUs, 9701 sequences) among rhizoctonia fungi, and ectomycorrhizal fungi from the Thelephoraceae (23 OTUs, 13297 sequences), Sebacinaceae (26 OTUs, 8420 sequences) and Inocybaceae (14 OTUs, 8261 sequences) (**Figure 1**). Besides, a number of other ectomycorrhizal taxa previously shown to associate with these orchid species were detected, including members of the Cortinariaceae (8 OTUs, 2709 sequences), Tuberaceae (1 OTU, 376 sequences), and Pezizaceae (4 OTUs, 97 sequences). Additionally, a number of ectomycorrhizal or possibly endophytic fungi belonging to the Psathyrellaceae, Tricholomataceae, and Russulaceae were only sporadically observed (**Figure 1**).

**FIGURE 2:**
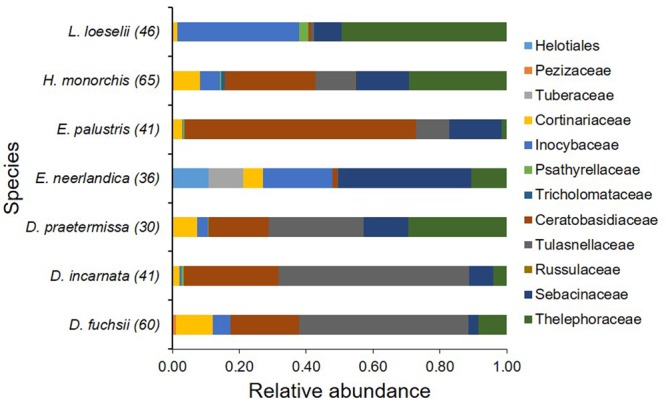
Relative abundance of orchid mycorrhizal families associating with the seven orchid species that co-occur in dune slack communities. Relative abundance was calculated as the proportion of DNA sequences in each species assigned to each fungal family. The number of OTUs retrieved for each species is given between brackets.

Mycorrhizal communities associating with each species were diverse (**Figure 2**) (average number of OTUs per species per population: 37.8 ± 7.6, range: 23–48) and all sampled individuals associated with multiple fungi simultaneously. The relative abundances of the fungal genera differed between species (**Figure 2**). Members of the Tulasnellaceae and Ceratobasidiaceae were retrieved from all sampled species, but only in very low abundances in *E. neerlandica* and *L. loeselii* (**Figure 2**). *Tulasnella* fungi were the dominant community members of *Dactylorhiza* species, whereas members of the Ceratobasidiaceae fungi dominated the mycorrhizal community associating with *E. palustris* (**Figure 2**), none of which clustered with ectomycorrhizal Ceratobasidiaceae taxa (*sensu* Veldre et al., 2013). Members of the Inocybaceae reached high relative abundances in *L. loeselii* and *E. neerlandica* and to a lesser extent in *H. monorchis*. *Tuber* reached high relative abundances in *E. neerlandica*, but was absent in all other species. Finally, members of the Sebacinaceae reached high relative abundances in *E. neerlandica*, whereas members of the Thelephoraceae had the highest relative abundances in *L. loeselii*.

Because *Tulasnella* strains have very derived ITS sequences (Dearnaley et al., 2012), additional analyses were performed using primers specifically designed for ITS detection of other *Tulasnella* species, in particular *Tulasnella calospora*. This strain appeared to be a major fungal associate in *L. loeselii* (Ouanphanivanh et al., 2008; Illyés et al., 2009) and was sporadically observed in *Dactylorhiza* (Jacquemyn et al., 2012, 2016b). However, while these primers successfully detected positive controls, the *Liparis* and *Dactylorhiza* root samples displayed no detectable strains of the *T. calospora* clade (**Supplementary Figure S1**).

**FIGURE 3:**
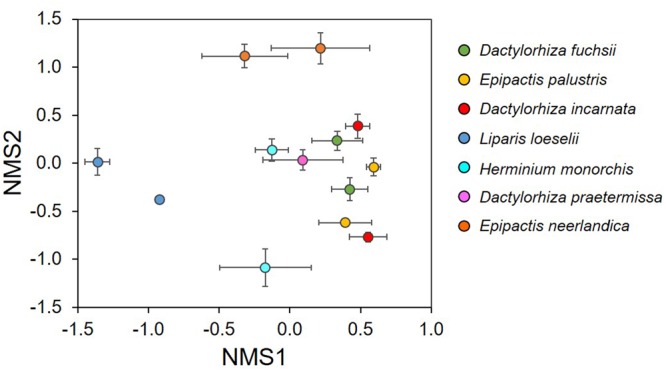
Non-metric multidimensional scaling (NMDS) plot illustrating differences in mycorrhizal communities between seven orchid species that co-occur in dune slack communities. Each data point represents the average scores of five individual plants for each species and population.

The NMDS ordination showed that the investigated orchid species associated with distinctive mycorrhizal communities (**Figure 3**). The mycorrhizal communities associating with *E. neerlandica* and *L. loeselii* were clearly different from the other species, which tended to cluster together in the central part of the plot. The PERMANOVA analysis confirmed that community composition of mycorrhizal fungi differed significantly between the investigated orchid species (*R*^2^ = 0.34, pseudo-*F* = 3.81, *P* < 0.001). Species Indicator Analysis revealed 24 indicator species. *Dactylorhiza* species were significantly associated with *Tulasnella* spp. (members of the rhizoctonias). *E. neerlandica* was significantly associated with ectomycorrhizal *Tuber, Inocybe* and several *Thelephora* OTUs, whereas rhizoctonia *Ceratobasidium* was a significant indicator species for *E. palustris*. Finally, *L. loeselii* significantly associated with several ectomycorrhizal *Thelephora* and *Inocybe* OTUs.

### Isotope and Total N Analyses of Coexisting Orchids at Westhoek and Ter Yde

**FIGURE 4:**
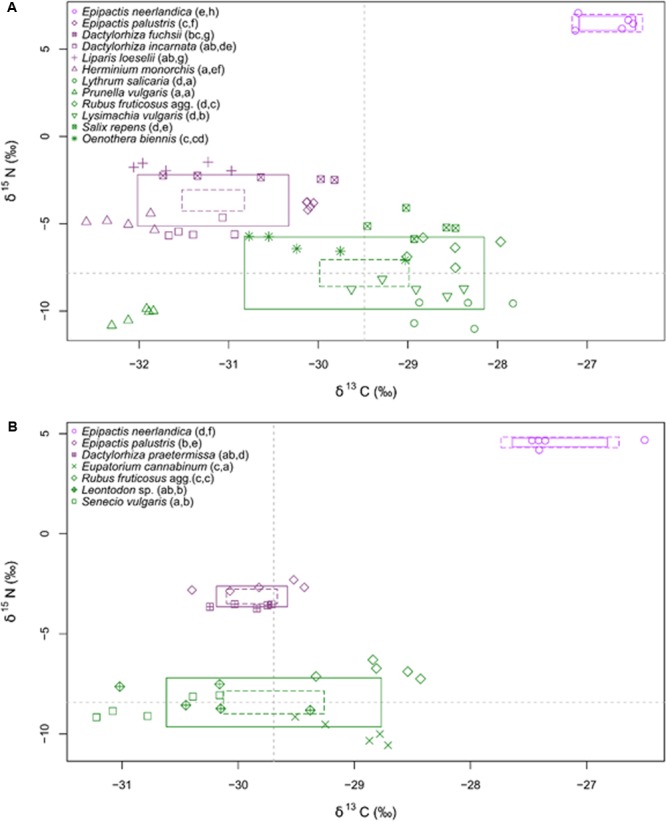
δ^13^C and δ^15^N values of plants at Westhoek **(A)** and Ter Yde **(B)**. Values for autotrophic non-orchid species are in green, and for orchid species in violet (with *E. neerlandica* in pink violet); *n* = 5 replicates per species. The boxes show the 95% confidence interval (dotted line) and standard deviation (continuous line) for each category. The gray dotted lines indicate the mean δ^13^C and δ^15^N values for non-orchids autotrophs. Different letters in brackets following the names indicate significantly different values for, respectively, δ^13^C and δ^15^N (*P* < 0.05).

At both sites, *E. neerlandica* was significantly more enriched in ^13^C and ^15^N than all other species (*P* < 0.001 for all comparisons), supporting a mixotrophic nutrition (**Figure 4**). δ^13^C values for the species collected at the two sites did not differ significantly (namely for *E. neerlandica, P* = 0.084; *E. palustris, P* = 0.227; and *Rubus fruticosus* agg., *P* = 0.342). δ^15^N values for *E. neerlandica* were significantly higher at Westhoek (6.48 ± 0.40; mean ± standard deviation) than at Ter Yde (4.57 ± 0.21; *P* < 0.001), whereas *E. palustris* showed lower values at Westhoek (-3.91 ± 0.20) compared to Ter Yde (-2.67 ± 0.22; *P* < 0.001), while *R. fruticosus* agg. had similar values at both sites (*P* = 0.365). Regarding autotrophic references, *Prunella vulgaris* at Westhoek had very low δ^13^C (-32.01 ± 0.19; **Figure 4A**) compared to the mean value for other autotrophic species (-28.98 ± 0.75). At Westhoek, all orchids except *E. neerlandica* were depleted in ^13^C relative to the autotrophic references except *P. vulgaris* (*P* < 0.05 for all comparisons). Notable exceptions were *D. fuchsii* and *E. palustris* that were not distinguishable from *Oenothera biennis* (*P* = 0.616; *P* = 1; **Figure 4A**). At Ter Yde, all orchids besides *E. neerlandica* were either depleted (compared to *Eupatorium cannabinum* and *R. fruticosus* agg., *P* < 0.05 for all comparisons) or enriched in ^13^C (compared to *Senecio vulgaris, P* < 0.05 for both comparisons; **Figure 4B**) or showed no difference in ^13^C (compared to *Leontodon* sp., *P* > 0.05 for both comparisons). As for ^15^N, the orchids outside *E. neerlandica* had higher δ^15^N than most of the autotrophic references (*P* < 0.05 for all comparisons) at both sites; the only exceptions were *D. incarnata* and *H. monorchis* at Westhoek that were not distinguishable from *O. biennis* and *Salix repens* for the former (*P* = 0.121; *P* = 0.998) and *Salix* sp. for the latter (*P* = 1).

**FIGURE 5:**
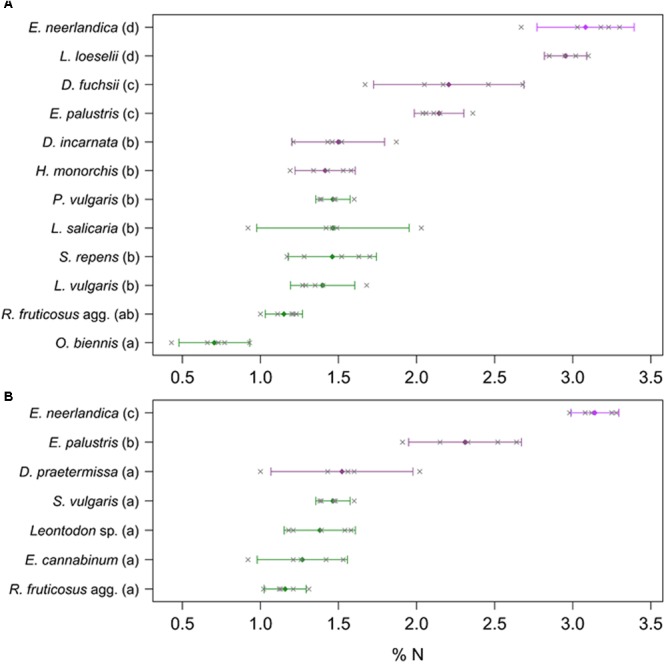
Total N content of plants at Westhoek **(A)** and Ter Yde **(B)**. Non-orchids are in green and orchids are in violet (with *E. neerlandica* in pink violet); *n* = 5 replicates per species. Colored diamonds represent mean values whereas gray crosses show the five corresponding measures. Bars represent 95% confidence intervals; different letters indicate statistically significant differences (*P* < 0.05).

Considering total N concentrations, the orchids fell in three distinct groups at Westhoek (**Figure 5A**): *E. neerlandica* and *L. loeselii* had the highest N concentrations (*P* < 0.001 for all comparisons with other species); *D. fuchsii* and *E. palustris* had intermediate N concentrations (*P* < 0.01 for all comparisons with other species); and all other orchids did not significantly differ from most autotrophic non-orchid species (*P* > 0.05 for all comparisons with other species except *O. biennis*). The situation was similar at Ter Yde with *E. neerlandica* having the highest N concentration (*P* < 0.001 for all comparisons; **Figure 5B**), *E. palustris* having intermediate N concentrations (*P* < 0.001 for all comparisons), whereas *D. praetermissa* and autotrophic references had similar N concentrations (*P* > 0.05 for all comparisons) lower than those of the two former species. Overall, all orchids tended to be enriched in ^15^N compared to surrounding autotrophic reference plants and four out of seven had higher total N concentrations, but only *E. neerlandica* also displayed the ^13^C enrichment characterizing mixotrophy.

## Discussion

### Rhizoctonia versus Ectomycorrhizal Fungi: A Continuum of Orchid-Fungus Associations in Dune Slacks

Our results showed that different orchid species co-occurring within dune slacks associated with diverse fungal communities. These fungi encompassed true rhizoctonia fungi (*sensu* Dearnaley et al., 2012), namely Tulasnellaceae and Ceratobasidiaceae (Serendipitaceae were absent), as well as various ectomycorrhizal clades (Thelephoraceae, Sebacinaceae, Inocybaceae, Tuberaceae, and Pezizaceae). The latter are likely supported by surrounding willows (*Salix repens*), which are ectomycorrhizal hosts to these taxa in dune ecosystems (Geml et al., 2014; Boonen et al., 2015) and which can provide them carbon. Their abundance in terms of reads and OTUs, given that they do not belong to usual contaminant fungi in soil samples, suggests that their presence has a true biological meaning. In *E. neerlandica* at least, the mycorrhizal status of these taxa can be supported since this species is phylogenetically close to, although ecologically isolated from *E. helleborine* (Jacquemyn et al., 2017). The latter commonly associates with ectomycorrhizal fungi (Bidartondo et al., 2004; Ogura-Tsujita and Yukawa, 2008; Těšitelová et al., 2012). Moreover, its isotopic values (see below) suggest that it belongs to mixotrophic species that receive organic matter from ectomycorrhizal fungi. In *L. loeselii*, the absence of rhizoctonia fungi also suggests that ectomycorrhizal fungi are the orchid’s mycorrhizal partners.

Surprisingly, we did not observe a clear separation between orchids harboring rhizoctonia and ectomycorrhizal fungi, but rather a ‘spectral’ continuum of mycorrhizal communities was found, challenging the idea of a strict dichotomy between these two types of orchids. Our molecular analyses showed that all investigated species associated with both rhizoctonia and ectomycorrhizal fungi, although the prevalence of each fungal guild differed among species. At one extreme, *E. neerlandica* was nearly devoid of rhizoctonia fungi, whereas *L. loeselii* was very poor in rhizoctonia fungi; the same trend is also supported by analyses of this species at other sites (Waud et al., 2017). This trend contrasts with previous results from fen habitats in Hungary (Illyés et al., 2005, 2009), perhaps because the latter were obtained after *in vitro* isolation of the fungus: this procedure often screens against slow-growing ectomycorrhizal fungi. At the other extreme, *Dactylorhiza* spp. predominantly associated with rhizoctonia fungi, which largely supports previous works (e.g., Rasmussen, 1995; Kristiansen et al., 2001; Bidartondo et al., 2004; Shefferson et al., 2008; Bailarote et al., 2012; Jacquemyn et al., 2012, 2016b). *E. palustris* also showed a preference for rhizoctonia fungi, mainly fungi from the Ceratobasidiaceae and to a lesser extent from the Tulasnellaceae (Bidartondo et al., 2004; Jacquemyn et al., 2016a). An intermediate situation where both fungal guilds were present was found in *H. monorchis* (whose mycorrhizal fungi were never studied previously to the best of our knowledge; Rasmussen, 1995).

The presence of ectomycorrhizal fungi in rhizoctonia-associated orchids has only been reported sporadically before the rise of high-throughput barcoding methods (e.g., Kristiansen et al., 2001; Shefferson et al., 2007; Jacquemyn et al., 2010, 2011; Liebel et al., 2015; Těšitelová et al., 2015) and it was suggested that their presence should not be neglected in analyses of partner diversity (Selosse et al., 2010). The rise of next-generation sequencing, which significantly enhanced the sensitivity of detection and the ability to handle samples that are colonized by multiple fungi, confirmed this trend (e.g., Jacquemyn et al., 2014, 2015; Oja et al., 2015). Still, the exact interaction of ectomycorrhizal fungi with rhizoctonia-associated orchids, in terms of root (or even cell) colonization, and their functional importance remains unclear. It can be questioned whether they are simple intercellular endophytes or whether they form intracellular pelotons similar to true mycorrhizal fungi in orchids. More detailed microscopic and physiological investigations are now needed to investigate whether ectomycorrhizal fungi in rhizoctonia-associated orchids are simple root commensalists or whether they bring nutrients, as can be expected from their roles in mixotrophic orchid species that exclusively associate with them.

### The Guild of Fungal Associates Does Not Always Predict Nutrition Type

Isotopic abundances showed that, although most orchids were enriched in ^15^N abundance and total N, ^13^C enrichment was only present in *E. neerlandica*, supporting the idea that it was the only mixotrophic orchid species based on these features. This species is the only one that grows at the edges of dune slacks, where the water table is probably lower than in dune slacks, and it was mostly colonized by ectomycorrhizal fungi of the Thelephoraceae, Tuberaceae, and Inocybaceae. Thus, *E. neerlandica* perfectly fits the mixotrophic syndrome described in the introduction. At the opposite, *D. fuchsii, D. incarnata* and *D. praetermissa, E. palustris* and *H. monorchis*, with total N content and ^13^C enrichment close to that of autotrophic non-orchid plants, fitted the syndrome of autotrophic orchids (see the possible reservations and current debates over the exact autotrophy in this syndrome in Introduction). Orchids that mostly associated with rhizoctonia fungi only tended to have slightly lower ^13^C enrichments as compared to non-orchid plants (discussed below), suggesting that ectomycorrhizal fungi did not contribute massively to their carbon budget. This is especially true for *H. monorchis* that, in spite of a high abundance of ectomycorrhizal fungi, turned out to be even significantly depleted in 13C as compared to autotrophic non-orchid plants.

The situation was less clear for *L. loeselii*, which mainly associated with members of the Thelephoraceae and Inocybaceae, with minor and infrequent occurrence of rhizoctonia fungi (see also Waud et al., 2017). Although its total N content was similarly high to that of the mixotrophic *E. neerlandica*, it was not enriched in ^13^C, and was in this respect similar to the orchids that mostly associated with rhizoctonia fungi. *L. loeselii* mycorrhizal partners are normally enriched in ^13^C as other ectomycorrhizal fungi (Kohzu et al., 1999), although we did not measure this in the investigated sites, so that no evidence for mixotrophy was found in *L. loeselii*. Thus, the investigated species set from dune slacks display a disconnection between the presence of ectomycorrhizal fungi and mixotrophy, as estimated from ^13^C and total N enrichment.

### The Evolutionary Lability of Nutritional Syndromes in Orchids

Our results further support previous results that have shown that *E. palustris* is autotrophic (Bidartondo et al., 2004; Hynson et al., 2013; Schiebold et al., 2017). The confirmation of autotrophy in *E. palustris* contrasts with the mixtrophy usually observed in other *Epipactis* spp. (Selosse et al., 2004; Bidartondo et al., 2004; Gonneau et al., 2014; see Hynson et al., 2013 for review). The genus *Epipactis* therefore represents another example of orchid genera that encompass both mixo- and autotrophic species, such as also observed in *Cymbidium* (Ogura-Tsujita et al., 2012) and *Neottia* (Těšitelová et al., 2015). These genera are relevant to study the shift to mixotrophy (since autotrophy is likely the ancestral state), and the possibility of reversions from mixo- to autotrophy.

Our data confirm previous reports that have shown the presence of ectomycorrhizal fungi in orchids that primarily associate with rhizoctonia-like fungi. Even if their exact role and influence remain unclear in autotrophic orchids, they may potentially indicate a first step in the evolution of mixotrophy assisted by ectomycorrhizal fungi, and suggest that the evolution to mixotrophy occurs in two consecutive steps. In such a scenario, ectomycorrhizal fungi would first gradually replace rhizoctonia fungi as mycorrhizal partners, without necessarily providing carbon, as suggested by the investigated *L. loeselii*. Subsequently, their indirect access to substantial nutrient resources from tree photosynthesis may allow the evolution of mixotrophy. However, whether ectomycorrhizal fungi in rhizoctonia-associated orchids represent a true predisposition remains to be assessed in other orchid lineages where mixotrophy has evolved. In this framework, the genus *Epipactis* represents, among others, an interesting taxon where the mix of species with different trophic modes and different symbionts, rhizoctonia and/or ectomycorrhizal fungi, offers possibilities to test this scenario thanks to phylogenetic reconstructions.

## Conclusion

Overall, our data demonstrate that co-occurring orchid species have distinctive mycorrhizal communities and indicate that the association with rhizoctonia fungi versus ectomycorrhizal fungi is not an alternative, but rather a continuum between extremes that exists within a given ecosystem. Moreover, our combined analysis of isotopes and fungal communities revealed that the presence of ectomycorrhizal fungi does not entail evidence for mixotrophy, at least in terms of ^13^C and N content, as shown by *L. loeselii*. There are thus intermediates between the two extreme syndromes, namely rhizoctonia-associated orchids, often considered autotrophic, versus mixotrophic orchids associated with ectomycorrhizal fungi. This questions whether a low level of colonization by ectomycorrhizal fungi in rhizoctonia-associated orchids may predispose to the evolutionary transition to an association with ectomycorrhizal fungi in some orchid phyla. Our results therefore call for a more precise understanding of the nature and physiological significance of ectomycorrhizal fungi present within rhizoctonia-associated orchids.

## Author Contributions

HJ, RB, and M-AS designed the experiment, HJ and RB performed the field work, MW performed the molecular analyses, FL, P-EC, and AR performed the isotope analyses, HJ, MW, and FL performed the statistical analyses, HJ and M-AS wrote the first draft of the manuscript, all authors read and approved the final draft.

## Supplementary Material

The Supplementary Material for this article can be found online at: http://journal.frontiersin.org/article/10.3389/fpls.2017.01497/full#supplementary-material

FIGURE S1 Gel results from PCR analyses conducted for verification of *Tulasnella* species presence/absence in **(A)** *Liparis loeselii* and **(B)** *Dactylorhiza* root samples using an enhanced version of the *Tulasnella* specific primer combination for ITS (namely ITS1ngs/ITS4-Tul2, described in Oja et al., 2015). Similar results were obtained using the primer pair ITS1/ITS4-Tul (data not shown). Expected *Tulasnella* species band size range from 600 to 1200 bp based on previous experiments. PCR reactions were conducted in duplicated 25 μl reactions containing 5 ng DNA extract, 1 U Titanium Taq (Takara, Bio, United States), 2 mM dNTPs, and 20 μM primer combination ITS1ngs/ ITS4-Tul2. The amplification program was as follows: 2 min at 95°C, followed by 30 cycles of 30 s at 95°C, 30 s at 55°C, 1 min at 72°C and a final step at 72°C for 10 min. Duplicated PCR reactions were pooled and 5 μl of pooled PCR product was checked for amplification by separation on 1.5% agarose gel. Lane components for **(A)** are as follows: MM, SmartLadder reference (Eurogentec, Belgium); Lane 1, negative control – no template DNA; Lane 2, negative fungal DNA control – *Mortierella isabellina* isolate UAMH 5163; Lane 3, positive control – *Tulasnella calospora* isolate CBS 573.83; Lanes 4–5, non-*Tulasnella* mycorrhizal control – *Salix repens* root samples; Lanes 6–15, *Liparis loeselii* root samples. Lane components for *Dactylorhiza* species **(B)** are as follows: MM, SmartLadder reference (Eurogentec, Belgium); Lane 1, negative control – no template DNA; Lane 2, negative fungal DNA control – *Mortierella isabellina* isolate UAMH 5163; Lane 3, positive control – *Tulasnella calospora* isolate CBS 573.83; Lane 4, positive control – *Tulasnella* sp. isolate CBS 482.93; Lane 5, positive control – *Tulasnella* sp. isolate CBS 487.93; Lane 6, positive control – *Tulasnella* sp. isolate CBS 606.93; Lanes 7–8, non-*Tulasnella* mycorrhizal control – *Salix repens* root samples; Lanes 9–13, *Dactylorhiza fuchsii* (Westhoek) root samples; Lanes 14–18, *Dactylorhiza incarnata* (Westhoek) root samples; Lanes 19–23, *Dactylorhiza fuchsii* (Ter Yde) root samples; Lanes 24–28, *Dactylorhiza incarnata* (Ter Yde) root samples.

## Conflict of Interest Statement

The authors declare that the research was conducted in the absence of any commercial or financial relationships that could be construed as a potential conflict of interest.
