# Supplementary figures and images for: Mycorrhizal Associations and Trophic Modes in Coexisting Orchids: An Ecological Continuum between Auto- and Mixotrophy
Source: Front Plant Sci. 2017 Aug 29;8:1497. doi: 10.3389/fpls.2017.01497 (PMC5583604; doi:10.3389/fpls.2017.01497)

## Slide 1
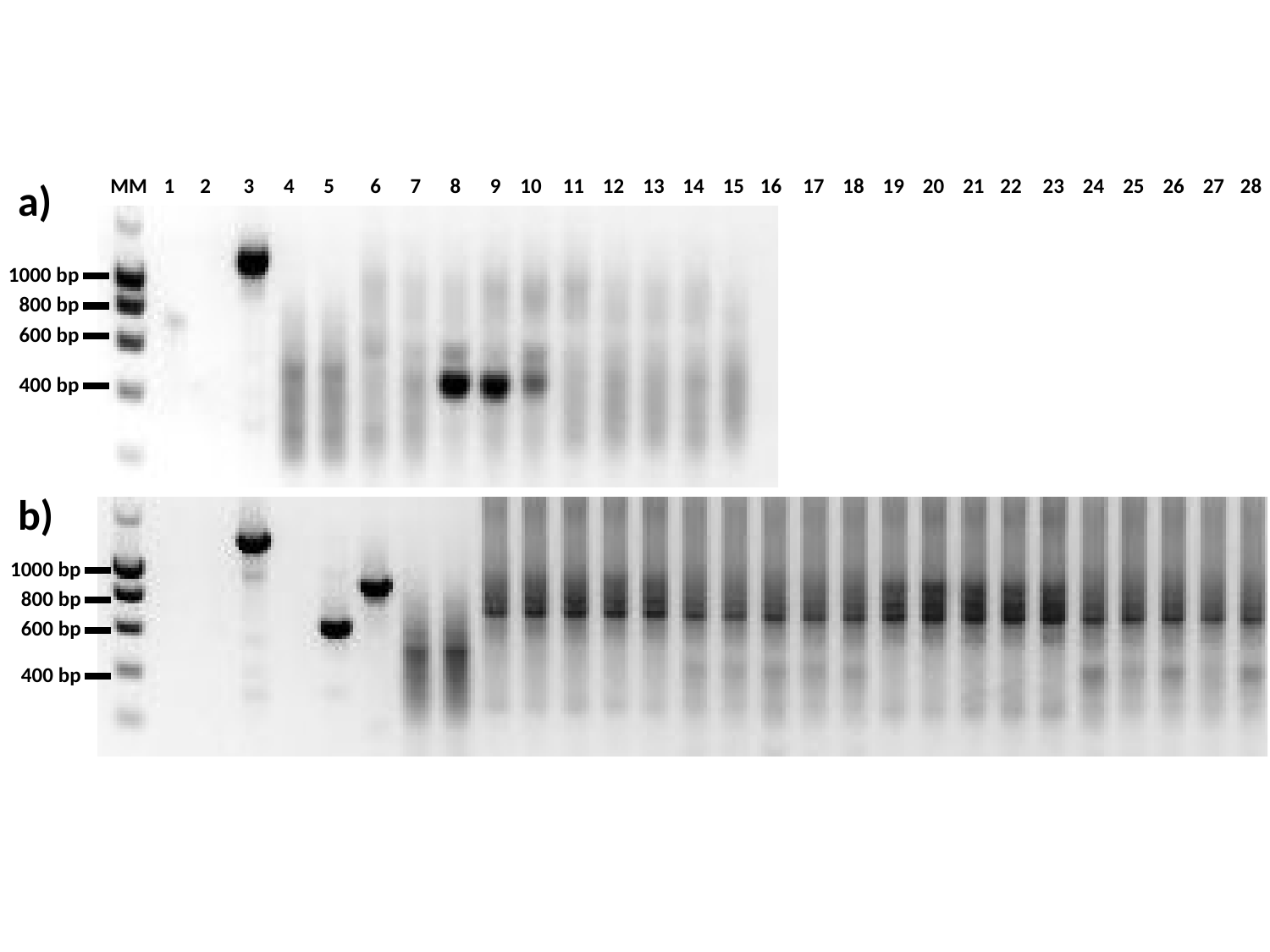

MM
1
2
3
4
5
6
7
8
9
10
11
12
13
14
15
16
17
18
19
20
21
22
23
24
25
26
27
28
a)
1000 bp
800 bp
600 bp
400 bp
b)
1000 bp
800 bp
600 bp
400 bp

Supplement: FIGURE S1 — Gel results from PCR analyses conducted for verification of Tulasnella species presence/absence in (A) Liparis loeselii and (B) Dactylorhiza root samples using an enhanced version of the Tulasnella specific primer combination for ITS (namely ITS1ngs/ITS4-Tul2, described in Oja et al., 2015). Similar results were obtained using the primer pair ITS1/ITS4-Tul (data not shown). Expected Tulasnella species band size range from 600 to 1200 bp based on previous experiments. PCR reactions were conducted in duplicated 25 μl reactions containing 5 ng DNA extract, 1 U Titanium Taq (Takara, Bio, United States), 2 mM dNTPs, and 20 μM primer combination ITS1ngs/ ITS4-Tul2. The amplification program was as follows: 2 min at 95°C, followed by 30 cycles of 30 s at 95°C, 30 s at 55°C, 1 min at 72°C and a final step at 72°C for 10 min. Duplicated PCR reactions were pooled and 5 μl of pooled PCR product was checked for amplification by separation on 1.5% agarose gel. Lane components for (A) are as follows: MM, SmartLadder reference (Eurogentec, Belgium); Lane 1, negative control – no template DNA; Lane 2, negative fungal DNA control – Mortierella isabellina isolate UAMH 5163; Lane 3, positive control – Tulasnella calospora isolate CBS 573.83; Lanes 4–5, non-Tulasnella mycorrhizal control – Salix repens root samples; Lanes 6–15, Liparis loeselii root samples. Lane components for Dactylorhiza species (B) are as follows: MM, SmartLadder reference (Eurogentec, Belgium); Lane 1, negative control – no template DNA; Lane 2, negative fungal DNA control – Mortierella isabellina isolate UAMH 5163; Lane 3, positive control – Tulasnella calospora isolate CBS 573.83; Lane 4, positive control – Tulasnella sp. isolate CBS 482.93; Lane 5, positive control – Tulasnella sp. isolate CBS 487.93; Lane 6, positive control – Tulasnella sp. isolate CBS 606.93; Lanes 7–8, non-Tulasnella mycorrhizal control – Salix repens root samples; Lanes 9–13, Dactylorhiza fuchsii (Westhoek) root samples; Lanes 14–18, Dacty [file Presentation_1.PPTX]
