# Supplementary material for: Mycorrhizal Associations and Trophic Modes in Coexisting Orchids: An Ecological Continuum between Auto- and Mixotrophy
Source: Front Plant Sci. 2017 Aug 29;8:1497. doi: 10.3389/fpls.2017.01497 (PMC5583604; doi:10.3389/fpls.2017.01497)
Supplement: Supplementary file 2 [file Table_1.DOCX]

**Table S1** List of operational taxonomic units (OTUs)^a^ corresponding to orchid-associating mycorrhizal families^b^ discovered in this study. OTUs are ordered in decreasing number of sequences.

| OTU | # sequences | Accession number | Family | Closest Blast match | Length | Score | Expected | Percent |
| --- | --- | --- | --- | --- | --- | --- | --- | --- |
| 2 | 20393 | KY083559 | Ceratobasidiaceae | Uncultured Ceratobasidiaceae clone J3M-2A | 250 | 250 | 1.10E-126 | 100.00% |
| 7 | 6023 | KY083563 | Inocybaceae | Uncultured *Inocybe* clone Mt_7_asal05_02 | 250 | 250 | 1.10E-126 | 100.00% |
| 13 | 4452 | KY083567 | Tulasnellaceae | Uncultured Tulasnellaceae clone LP54-4T | 250 | 247 | 5.20E-125 | 99.60% |
| 9 | 2886 | KY083565 | Thelephoraceae | Uncultured Thelephorales clone | 250 | 250 | 1.10E-126 | 100.00% |
| 3 | 2620 | KY083560 | Thelephoraceae | Uncultured Thelephoraceae clone | 250 | 232 | 1.10E-116 | 97.60% |
| 1 | 2284 | KY083558 | Thelephoraceae | *Tomentella* sp. Sc-S | 250 | 247 | 5.20E-125 | 99.60% |
| 5 | 2243 | KY083561 | Thelephoraceae | Uncultured *Tomentella* | 250 | 244 | 2.40E-123 | 99.20% |
| 33 | 2094 | KY083576 | Tulasnellaceae | Uncultured Tulasnellaceae clone Z15 | 240 | 185 | 1.51E-90 | 92.10% |
| 1187 | 1879 | KY083664 | Sebacinaceae | Uncultured *Sebacina* mycobiont of *Riccardia palmata* | 250 | 238 | 5.20E-120 | 98.40% |
| 36 | 1701 | KY083578 | Sebacinaceae | Uncultured *Sebacina* isolate TUB 019439 clone H9 | 250 | 241 | 1.10E-121 | 98.80% |
| 45 | 1340 | KY083581 | Sebacinaceae | Uncultured *Sebacina* isolate B.7657.1 | 250 | 250 | 1.10E-126 | 100.00% |
| 64 | 1222 | KY083593 | Tulasnellaceae | Uncultured Tulasnellaceae clone LP73-6T | 250 | 250 | 1.10E-126 | 100.00% |
| 57 | 1072 | KY083588 | Cortinariaceae | Uncultured *Cortinarius* clone AR1327 | 250 | 247 | 5.20E-125 | 99.60% |
| 18 | 1064 | KY083569 | Inocybaceae | *Inocybe* cf. *squarrosoannulata* CLC1375 | 250 | 247 | 5.20E-125 | 99.60% |
| 6 | 1002 | KY083562 | Thelephoraceae | Uncultured *Tomentella* | 250 | 250 | 1.10E-126 | 100.00% |
| 88 | 745 | MF567578 | Tulasnellaceae | Uncultured Tulasnellaceae clone CF122 | 250 | 235 | 2.40E-118 | 98.00% |
| 1610 | 707 | KY083678 | Cortinariaceae | Uncultured *Cortinarius* clone AR1635 | 250 | 238 | 5.20E-120 | 98.40% |
| 93 | 638 | KY083601 | Sebacinaceae | Uncultured *Sebacina* clone 10361 | 249 | 239 | 1.40E-120 | 98.80% |
| 76 | 637 | MF567576 | Sebacinaceae | Uncultured *Sebacina* clone M8T5P4 | 251 | 244 | 2.40E-123 | 99.20% |
| 19 | 584 | KY083570 | Psathyrellaceae | Coprinopsis pachyderma voucher Ulje 1273 | 250 | 250 | 1.10E-126 | 100.00% |
| 30 | 562 | KY083575 | Cortinariaceae | Uncultured *Cortinarius* clone AR1224 | 250 | 250 | 1.10E-126 | 100.00% |
| 84 | 549 | MF567577 | Thelephoraceae | Uncultured ectomycorrhiza (Thelephoraceae) | 250 | 250 | 1.10E-126 | 100.00% |
| 16 | 496 | KY083568 | Sebacinaceae | Uncultured *Sebacina* mycobiont of *Calamagrostis epigeios* | 250 | 244 | 2.40E-123 | 99.20% |
| 11 | 462 | KY083566 | Incertae sedis | Uncultured Helotiales isolate 492 sequence | 250 | 250 | 1.10E-126 | 100.00% |
| 56 | 447 | KY083587 | Thelephoraceae | Uncultured ectomycorrhiza (Thelephoraceae) | 250 | 250 | 1.10E-126 | 100.00% |
| 92 | 444 | MF567579 | Tulasnellaceae | Uncultured Tulasnellaceae clone J1C-1A | 250 | 242 | 3.10E-122 | 98.40% |
| 77 | 415 | KY083597 | Sebacinaceae | Uncultured *Sebacina* isolate TUB 019377 | 126 | 107 | 3.46E-47 | 95.20% |
| 184 | 388 | KY083617 | Inocybaceae | *Inocybe umbrinella* voucher 4375 | 250 | 250 | 1.10E-126 | 100.00% |
| 1102 | 378 | MF567595 | Tulasnellaceae | Uncultured Tulasnellaceae clone Z15 | 250 | 183 | 1.95E-89 | 90.80% |
| 125 | 376 | MF567582 | Tuberaceae | Uncultured Tuberaceae clone | 251 | 244 | 2.40E-123 | 99.20% |
| 24 | 357 | KY083572 | Thelephoraceae | Uncultured *Tomentella* | 250 | 247 | 5.20E-125 | 99.60% |
| 197 | 331 | KY083619 | Sebacinaceae | Uncultured Sebacinaceae clone OTU-0829 | 250 | 244 | 2.40E-123 | 99.20% |
| 27 | 329 | KY083573 | Thelephoraceae | Uncultured Thelephoraceae clone 8 | 250 | 247 | 5.20E-125 | 99.60% |
| 35 | 329 | KY083577 | Cortinariaceae | *Hebeloma salicicola* voucher Henry J. Beker:HJB13087 | 250 | 250 | 1.10E-126 | 100.00% |
| 96 | 326 | MF567580 | Tulasnellaceae | Uncultured Tulasnellaceae clone 4-22-1A | 240 | 198 | 8.96E-98 | 94.20% |
| 62 | 211 | KY083591 | Sebacinaceae | Uncultured *Sebacina* mycobiont of *Phleum pratense* | 250 | 250 | 1.10E-126 | 100.00% |
| 127 | 209 | KY083605 | Inocybaceae | *Inocybe vulpinella* strain 3918 | 250 | 250 | 1.10E-126 | 100.00% |
| 75 | 204 | KY083596 | Ceratobasidiaceae | *Ceratobasidium* sp. AG-I isolate HuN-4-1 | 250 | 250 | 1.10E-126 | 100.00% |
| 183 | 198 | KY083616 | Thelephoraceae | Uncultured *Tomentella* clone HV_D1_4a | 249 | 224 | 3.20E-112 | 96.80% |
| 154 | 191 | MF567583 | Inocybaceae | *Inocybe* sp. OTU288 | 250 | 231 | 4.10E-116 | 97.60% |
| 107 | 174 | MF567581 | Inocybaceae | Uncultured *Inocybe* clone AR1662 | 250 | 250 | 1.10E-126 | 100.00% |
| 191 | 117 | KY083618 | Sebacinaceae | Uncultured *Sebacina* isolate TUB 019459 clone H4 | 250 | 250 | 1.10E-126 | 100.00% |
| 53 | 115 | KY083585 | Sebacinaceae | Uncultured *Sebacina* mycobiont of *Dipsacus sylvestris* | 250 | 250 | 1.10E-126 | 100.00% |
| 180 | 99 | KY083615 | Thelephoraceae | Uncultured Thelephoraceae | 250 | 237 | 1.90E-119 | 98.40% |
| 233 | 99 | MF567585 | Sebacinaceae | Uncultured *Sebacina* clone KV13d_17_A03 | 137 | 107 | 3.46E-47 | 92.70% |
| 28 | 89 | KY083574 | Sebacinaceae | Uncultured *Sebacina* isolate TUB 019459 clone H3 | 247 | 175 | 5.47E-85 | 90.70% |
| 204 | 75 | MF567584 | Sebacinaceae | *Sebacina* sp. Seb15I | 117 | 99 | 9.36E-43 | 94.90% |
| 352 | 75 | MF567587 | Sebacinaceae | Uncultured *Sebacina* mycobiont of *Glechoma hederacea* | 255 | 204 | 4.10E-101 | 93.70% |
| 211 | 61 | KY083621 | Thelephoraceae | Uncultured *Tomentella* | 250 | 244 | 2.40E-123 | 99.20% |
| 353 | 61 | KY083635 | Pezizaceae | Uncultured Pezizaceae clone | 250 | 206 | 3.20E-102 | 94.40% |
| 1385 | 61 | MF567599 | Inocybaceae | Uncultured *Inocybe* genomic DNA | 252 | 245 | 6.70E-124 | 99.20% |
| 8 | 57 | KY083564 | Inocybaceae | Uncultured *Inocybe* isolate MBN0213_15 | 249 | 246 | 1.90E-124 | 99.60% |
| 249 | 55 | KY083626 | Thelephoraceae | Uncultured Thelephoraceae clone | 241 | 203 | 1.50E-100 | 95.00% |
| 295 | 49 | MF567586 | Sebacinaceae | Uncultured *Sebacina* isolate TUB 019459 clone H3 | 250 | 250 | 1.10E-126 | 100.00% |
| 291 | 48 | KY083629 | Sebacinaceae | Uncultured *Sebacina* isolate TUB 019358 | 250 | 250 | 1.10E-126 | 100.00% |
| 304 | 40 | KY083630 | Inocybaceae | Uncultured *Inocybe* clone Mt_7_asal05_02 | 250 | 237 | 1.90E-119 | 98.40% |
| 306 | 36 | KY083631 | Tricholomataceae | Uncultured *Calyptella* clone IVP5-46 | 250 | 250 | 1.10E-126 | 100.00% |
| 59 | 35 | KY083589 | Ceratobasidiaceae | Uncultured ectomycorrhiza (Ceratobasidiaceae) 4099 | 250 | 247 | 5.20E-125 | 99.60% |
| 329 | 35 | KY083632 | Thelephoraceae | Uncultured *Thelephora* | 250 | 250 | 1.10E-126 | 100.00% |
| 142 | 32 | KY083608 | Tricholomataceae | Tricholomataceae sp. HD-2014 isolate DO74 | 203 | 147 | 2.01E-69 | 91.10% |
| 377 | 30 | KY083636 | Pezizaceae | Pezizaceae sp. | 239 | 140 | 1.57E-65 | 86.60% |
| 432 | 27 | KY083639 | Psathyrellaceae | *Coprinellus verrucispermus* strain SZMC-NL-2146 | 250 | 250 | 1.10E-126 | 100.00% |
| 382 | 26 | KY083637 | Thelephoraceae | Uncultured *Tomentella* | 250 | 250 | 1.10E-126 | 100.00% |
| 472 | 25 | KY083641 | Inocybaceae | *Inocybe tjallingiorum* | 250 | 250 | 1.10E-126 | 100.00% |
| 996 | 25 | KY083661 | Ceratobasidiaceae | Rhizoctonia sp. AU31 | 252 | 242 | 3.10E-122 | 98.80% |
| 49 | 24 | KY083584 | Sebacinaceae | Uncultured *Sebacina* isolate TUB 019460 clone 8B | 250 | 250 | 1.10E-126 | 100.00% |
| 173 | 24 | KY083613 | Tricholomataceae | Tricholomataceae sp. HD-2014 isolate DO74 | 203 | 150 | 4.32E-71 | 91.60% |
| 852 | 23 | KY083653 | Thelephoraceae | Uncultured Thelephoraceae clone | 250 | 246 | 1.90E-124 | 99.60% |
| 603 | 22 | MF567590 | Tulasnellaceae | Uncultured Tulasnellaceae clone CF361 | 250 | 226 | 2.40E-113 | 96.80% |
| 39 | 20 | KY083579 | Cortinariaceae | *Hebeloma dunense* voucher KRAM:F57434 | 250 | 250 | 1.10E-126 | 100.00% |
| 410 | 20 | MF567589 | Thelephoraceae | Uncultured ectomycorrhiza (Tomentella) | 250 | 250 | 1.10E-126 | 100.00% |
| 95 | 18 | KY083602 | Inocybaceae | *Inocybe arenicola* voucher RC_GB99_014 | 250 | 250 | 1.10E-126 | 100.00% |
| 545 | 18 | KY083643 | Sebacinaceae | Uncultured Sebacinaceae clone 5S3.12.S04 | 251 | 177 | 4.23E-86 | 90.40% |
| 859 | 17 | KY083656 | Thelephoraceae | Uncultured Thelephoraceae | 250 | 230 | 1.50E-115 | 97.60% |
| 1358 | 17 | KY083672 | Thelephoraceae | Uncultured *Tomentella* | 250 | 244 | 2.40E-123 | 99.20% |
| 345 | 16 | KY083634 | Sebacinaceae | Uncultured *Sebacina* clone 10513 | 250 | 250 | 1.10E-126 | 100.00% |
| 80 | 15 | KY083599 | Russulaceae | Uncultured *Lactarius* clone d43_1Lac | 250 | 250 | 1.10E-126 | 100.00% |
| 46 | 13 | KY083582 | Thelephoraceae | Uncultured *Tomentella* clone AR1144 | 250 | 250 | 1.10E-126 | 100.00% |
| 130 | 13 | KY083606 | Sebacinaceae | Uncultured *Sebacina* isolate 11042 | 250 | 247 | 5.20E-125 | 99.60% |
| 251 | 11 | KY083627 | Sebacinaceae | Uncultured *Sebacina* isolate TUB 019453 clone D1 | 253 | 203 | 1.50E-100 | 93.70% |
| 740 | 11 | KY083647 | Sebacinaceae | Uncultured *Sebacina* mycobiont of *Trifolium thalii* clone | 251 | 194 | 1.50E-95 | 92.80% |
| 511 | 10 | KY083642 | Thelephoraceae | Uncultured Thelephoraceae clone | 250 | 244 | 2.40E-123 | 99.20% |
| 1210 | 10 | KY083665 | Tulasnellaceae | Uncultured Tulasnellaceae clone OmiA/P120_C_30 | 251 | 247 | 5.20E-125 | 99.60% |
| 671 | 9 | MF567591 | Cortinariaceae | *Cortinarius infractus* voucher 6140 | 252 | 238 | 5.20E-120 | 98.40% |
| 229 | 8 | KY083624 | Cortinariaceae | *Cortinarius saturninus* strain ME12_F2 | 251 | 247 | 5.20E-125 | 99.60% |
| 825 | 8 | MF567592 | Tulasnellaceae | Uncultured Tulasnellaceae isolate P350 | 250 | 241 | 1.10E-121 | 98.80% |
| 1020 | 8 | KY083662 | Psathyrellaceae | *Coprinopsis rugosobispora* | 250 | 250 | 1.10E-126 | 100.00% |
| 933 | 7 | KY083658 | Tricholomataceae | *Flagelloscypha minutissima* strain KRP56-6 | 250 | 247 | 5.20E-125 | 99.60% |
| 20 | 6 | KY083571 | Sebacinaceae | Uncultured Sebacinaceae clone IIK1-18 | 251 | 247 | 5.20E-125 | 99.60% |
| 333 | 6 | KY083633 | Inocybaceae | Uncultured *Inocybe* clone WD_S1_9_47 | 236 | 233 | 3.10E-117 | 99.60% |
| 853 | 5 | KY083654 | Ceratobasidiaceae | Uncultured Ceratobasidium | 103 | 79 | 1.27E-31 | 92.20% |
| 868 | 5 | MF567593 | Psathyrellaceae | *Psathyrella prona* isolate 13147 | 250 | 247 | 5.20E-125 | 99.60% |
| 930 | 4 | MF567594 | Ceratobasidiaceae | Uncultured Ceratobasidiaceae clone OTU-0436 | 250 | 250 | 1.10E-126 | 100.00% |
| 1278 | 4 | MF567596 | Thelephoraceae | *Tomentella* sp. 3 CG-2012 | 248 | 227 | 6.60E-114 | 97.20% |
| 364 | 3 | MF567588 | Ceratobasidiaceae | *Heteroacanthella acanthophysa* voucher KC925 | 250 | 195 | 4.17E-96 | 92.80% |
| 658 | 3 | KY083646 | Pezizaceae | Uncultured Pezizaceae clone | 250 | 223 | 1.10E-111 | 96.40% |
| 854 | 3 | KY083655 | Sebacinaceae | Uncultured *Sebacina* isolate TUB 019446 clone B3 | 250 | 244 | 2.40E-123 | 99.20% |
| 1281 | 3 | MF567597 | Inocybaceae | *Inocybe* sp. 16289 | 250 | 247 | 5.20E-125 | 99.60% |
| 1370 | 3 | MF567598 | Sebacinaceae | Uncultured *Sebacina* | 250 | 250 | 1.10E-126 | 100.00% |
| 1420 | 3 | KY083674 | Pezizaceae | Pezizaceae sp. | 230 | 135 | 9.42E-63 | 86.50% |
| 63 | 2 | KY083592 | Inocybaceae | *Inocybe diabolica* WTU F-063180 | 253 | 218 | 6.80E-109 | 95.70% |
| 82 | 2 | KY083600 | Cortinariaceae | Uncultured *Cortinarius* | 250 | 250 | 1.10E-126 | 100.00% |
| 1229 | 2 | KY083666 | Thelephoraceae | Uncultured *Tomentella* | 251 | 244 | 2.40E-123 | 99.20% |
| 1400 | 2 | MF567600 | Ceratobasidiaceae | *Rhizoctonia* sp. ATT213 18S | 250 | 225 | 8.80E-113 | 96.80% |

^a^ OTUs were defined based on a 3% sequence dissimilarity cut-off value.

^b^According to Dearnaley *et al* (2012).
